# Clinical and imaging features of patients with cerebral autosomal dominant arteriopathy with subcortical infarcts and leukoencephalopathy and cysteine-sparing NOTCH3 mutations

**DOI:** 10.1371/journal.pone.0234797

**Published:** 2020-06-18

**Authors:** Hyunjin Kim, Young-Min Lim, Eun-Jae Lee, Yeo Jin Oh, Kwang-Kuk Kim

**Affiliations:** Department of Neurology, Asan Medical Center, University of Ulsan College of Medicine, Seoul, Republic of Korea; Johns Hopkins School of Medicine, UNITED STATES

## Abstract

**Background:**

Characteristics of patients with cerebral autosomal dominant arteriopathy with subcortical infarcts and leukoencephalopathy (CADASIL) and cysteine-sparing NOTCH3 mutations are relatively unknown. This study compared clinical and imaging characteristics between patients with CADASIL and cysteine-sparing NOTCH3 mutations and those with CADASIL and cysteine-involving NOTCH3 mutations.

**Methods:**

We retrospectively reviewed medical records of patients with CADASIL admitted to the Asan Medical Center between September 1999 and September 2017. We compared clinical and brain magnetic resonance imaging (MRI) characteristics based on the presence or absence of cysteine-involving NOTCH3 gene mutations. We compared white matter change frequencies and grades in specific spatial regions between the groups according to age-related white matter change (ARWMC) scores. We evaluated the presence, number, and anatomical distributions of cerebral microbleeds according to the microbleed anatomical rating scale.

**Results:**

We reviewed data from 79 patients (55 cysteine-involving, 24 cysteine-sparing NOTCH3 mutations). Clinical symptoms and signs did not differ significantly between the groups. The white matter change frequency and ARWMC scores (adjusted for age and stroke risk factors) in the anterior temporal lobes were lower in cysteine-sparing patients than in cysteine-involving patients. Frequencies and grades of the other brain region’s white matter changes and cerebral microbleeds were similar between the groups.

**Conclusions:**

Patients with CADASIL and cysteine-sparing NOTCH3 mutations showed less involvement of the anterior temporal lobes in brain MRI than those with CADASIL and cysteine-involving NOTCH3 mutations, although both groups showed similar clinical characteristics.

## Introduction

Cerebral autosomal dominant arteriopathy with subcortical infarcts and leukoencephalopathy (CADASIL) is an inherited systemic arterial vessel disease that affects middle-aged adults and leads to disability and dementia [1]. In 1996, mutations in the NOTCH3 gene were identified as the cause of CADASIL [2]. Most NOTCH3 mutations affect the number of cysteines in the extracellular domain of the NOTCH3 receptor, causing protein misfolding and receptor aggregation in small arteries [3–5]. Such mutant protein aggregation leads to chronic subcortical ischemia and characteristic clinical symptoms [6]. The main symptoms of CADASIL in patients are migraine with aura, subcortical ischemic events, mood disturbances, apathy, and cognitive impairment [6].

Although numerous cysteine-altering NOTCH3 mutations are known to cause CADASIL, studies have reported on patients with CADASIL and cysteine-sparing NOTCH3 mutations [7–14]. The association between cysteine-sparing NOTCH3 mutations and CADASIL has also been partly recognized but remains controversial [15]. Besides, clinical and imaging characteristics of CADASIL with cysteine-sparing mutations have been reported only for individual cases. Therefore, the characteristics of patients with CADASIL and cysteine-sparing NOTCH3 mutations need to be investigated and compared to those of patients with cysteine-involving NOTCH3 mutations. The purpose of this study was to compare the clinical and imaging characteristics between patients with CADASIL and cysteine-sparing mutations and those with CADASIL and cysteine-involving mutations.

## Materials and methods

### Patients

We retrospectively reviewed medical records of patients with CADASIL admitted to the Asan Medical Center between September 1999 and September 2017. Patients with characteristic clinical symptoms and brain magnetic resonance imaging (MRI) features or a positive family history underwent NOTCH3 gene testing. CADASIL was confirmed by identifying a mutation in the NOTCH3 gene on chromosome 19p13. Our review comprised available clinical and brain MRI data, including demographics; clinical symptoms and signs such as headache, stroke, mood disturbance, cognitive impairment, stroke risk factors (previously diagnosed and treated hypertension or systolic/diastolic blood pressure ≥ 140/90 mmHg, previously diagnosed and treated diabetes mellitus or hemoglobin A1c ≥ 6.5, and current smoking); and results of genetic analyses. Headache was defined as a history of any type of headache occurring frequently for > 6 months. Stroke was defined as the acute onset of symptoms and/or clinical signs of focal (or, in specific cases, global) neurologic dysfunction, lasting for >24 hours, with no other causes than vascular origin [16]. Mood disturbance was defined as a history of depression or bipolar disorder for which drug therapy had been administered. Cognitive impairment was defined as a previous diagnosis of dementia by a neurologist or a Mini-Mental State Examination score < 23.

### Genetic analysis

Genomic DNA samples were isolated from peripheral blood using PUREGENE DNA isolation kits (Gentra, Minneapolis, MN, USA). Eleven exons (2–11 and 18) of *NOTCH3* and their intronic flanking sequences were amplified by polymerase chain reaction (PCR) using 11 primer sets. After amplification, the size and purity of the PCR products were verified by separation on 1.2% agarose gels in the presence of ethidium bromide. After that, DNA sequencing was performed using the primer sets used for PCR using the BigDye Terminator v3.1 Cycle SequencingReady Reaction Kit (Applied Biosystems, Foster City, CA, USA) according to the manufacturer’s instructions. Electrophoresis and analysis were performed on an ABI 3130XL genetic analyzer (Applied Biosystems). The pathogenicity of novel missense variants was predicted using the following bioinformatics tools: Polyphen-2 (http://genetics.bwh.harvard.edu/pph2), Mutation taster (http://mutationtaster.org), and Sorting intolerant from tolerant (SIFT) (http://sift.jcvi.org). Frequencies of each variant were evaluated using the gnomAD database (http://gnomad.broadinstitute.org).

### MRI analysis

Brain MRI was performed on a 1.5-T (Siemens Avanto, Siemens Medical Solutions, Malvern, PA, USA) or 3.0-T (Philips Achieva, Philips Medical Systems, Andover, MA, USA) MRI system and included axial T2, fluid attenuation inversion recovery (FLAIR) and gradient-echo T2*-weighted (GRE) images. FLAIR images were acquired from a fast spin-echo sequence with the following parameters: repetition time/echo time, 9000/100 ms; inversion time, 2500 ms; and matrix, 256 × 224. The GRE parameters included 5-mm thick slices, 2-mm inter-slice gaps, 20 axial slices, 250-mm fields of view, 400-ms repetition times, 30-ms echo times, 20° flip angles, and 256×192 matrices. White matter abnormalities were graded in T2/FLAIR images according to the age-related white matter change (ARWMC) scoring system as follows: 0, absent; 1, focal; 2, initially confluent; 3, diffuse involvement of lesions in the frontal, parieto-occipital, infratentorial, anterior temporal, and external capsule regions; and 0, absent; 1, one focal lesion of > 5 mm; 2, more than one lesion; and 3, confluent for lesions in the basal ganglia [17]. White matter change was defined as an ARWMC score ≥ 1. Cerebral microbleeds were investigated in GRE images according to the microbleed anatomical rating scale (MARS) [18]. Cerebral microbleeds were evaluated based on their presence, number, and anatomical distribution (infratentorial, deep, and lobar) and defined as well-defined hypointense lesions within the brain parenchyma with clear margins ranging from 2 to 10 mm. Two investigators (H. Kim and Y.J. Oh) blinded to clinical data determined ARWMC scores and MARS by consensus; a third investigator (E.-J. Lee) was consulted in cases of disagreement. Only left hemispheres were scored. An analysis comparing the scores from the hemispheres found that they were equivalent for all ARWMC and MARS scores (S1 Table).

### Statistical analysis

From a previous study that reported the ARWMC score of CADASIL [19], we acquired the anticipated difference of the mean ARWMC score of the anterior temporal area between the two groups (cysteine-involving vs. cysteine-sparing) as 0.8, standard deviation as 1.2, and enrollment ratio as 2:1. Assuming a significance level of 5% and a power of 80%, the calculated sample size was 79. The study period was set to the target sample size.

We expressed descriptive data as frequencies and percentages for categorical variables and as mean ± standard deviation or median [interquartile range] for continuous variables. We performed Student’s *t*-tests for normally distributed variables and chi-square or Fisher’s exact tests for two-group comparisons of categorical variables, as appropriate. We performed ordinal logistic regression analysis with adjustment for covariates including age, hypertension, diabetes mellitus, and smoking to evaluate the association between patient groups and scores. Because the ARWMC score ranged from 0 to 3 depending on the severity of white matter change, the ARWMC score was reported on an ordinal scale. MARS was categorized according to tertiles for analysis by ordinal logistic regression. We considered two-sided p values < 0.05 to indicate statistical significance and performed all statistical analyses using IBM SPSS Statistics for Windows^®^, version 21.0 (IBM Corp., Armonk, NY, USA).

### Ethics statement

The institutional review board of Asan Medical Center approved this study (2017–0773) and waived the need for informed consent based on the retrospective study design. The study was performed following the principles of the Declaration of Helsinki.

## Results

We reviewed data from a total of 80 patients from 71 families. First, we reviewed all NOTCH3 mutations to exclude polymorphisms. We identified 22 different cysteine-involving mutations from 55 patients and 6 different cysteine-sparing mutations from 25 patients. All mutations were missense mutations. Among them, nine missense variants (five cysteine-involving mutations: G460C, C245Y, C493S, G481C, C134R and four cysteine-sparing mutations: P572L, D239N, R75Q, G73S) had not been previously reported. We evaluated their pathogenicity using bioinformatics tools (Table 1). A cysteine-sparing variant, R75Q, was deemed a polymorphism by Polyphen-2, mutations taster, and SIFT. Thus, we excluded the patient with R75Q mutation from the analysis.

**Table 1:** Pathogenicity of novel missense variants according to bioinformatics data.

Missense variants	Polyphen-2 Score	Mutation taster	SIFT	gnomAD (allele frequency)
**Gly460Cys**	1.000	Disease-causing	Affected, 0.00	Variant not found
**Pro572Leu**	0.943	Disease-causing	Affected, 0.00	Variant:19:15298041G/A (0.0003, East Asian)
**Cys245Tyr**	1.000	Disease-causing	Affected, 0.00	Variant not found
**Cys493Ser**	1.000	Disease-causing	Affected, 0.00	Variant not found
**Asp239Asn**	0.974	Disease-causing	Affected, 0.01	Variant:19:15302643C/T (0.0001, East Asian)
**Gly481Cys**	0.984	Disease-causing	Affected, 0.03	Variant not found
**Arg75Gln**	0.603	Polymorphism	Tolerated, 0.29	Variant:19:15303304C/T (0.0017, East Asian)
**Cys134Arg**	1.000	Disease-causing	Affected, 0.00	Variant not found
**Gly73Ser**	0.999	Disease-causing	Affected, 0.03	Variant not found

Polyphen-2 (http://genetics.bwh.harvard.edu/pph2), Mutation taster (http://mutationtaster.org), Sorting intolerant from tolerant (SIFT) (http://sift.jcvi.org), gnomAD (http://gnomad.broadinstitute.org)

We analyzed data from a total of 79 patients with CADASIL (55 and 24 with cysteine-involving and cysteine-sparing mutations, respectively). All patients were Korean, with a mean age at the time of assessment of 53.3 ± 12.0 years. Almost half of the patients were men (48.1%). The most common mutation was a cysteine-sparing R75P (18/79 [22.8%]), followed by cysteine-involving R544C (12/79 [15.2%]). Table 2 shows the clinical and imaging characteristics of patients with CADASIL. The ages at assessment, sex, and prevalence of stroke risk factors did not differ significantly between patients with cysteine-involving and cysteine-sparing NOTCH3 mutations. All clinical symptoms and signs including headache, stroke, mood disturbance, and cognitive impairment manifested similarly in both patient groups. Brain MRIs showed a high prevalence of white matter change in both groups, except for a higher rate of anterior temporal lobe hyperintensities in the cysteine-involving group than in the cysteine-sparing group (67.3%, 25.0%, respectively, p = 0.001). Total and regional frequencies of cerebral microbleeds were comparable between the groups.

**Table 2:** Clinical and imaging characteristics of patients with CADASIL according to cysteine mutation type.

	Cysteine-involving (n = 55)	Cysteine-sparing (n = 24)	p-value
**Age at assessment (y), mean ± SD**	52.1 ± 12.5	56.0 ± 10.3	0.185
**Men, number (%)**	28 (51.9)	10 (38.5)	0.261
**Stroke risk factors, number (%)**			
Hypertension	13 (23.6)	5 (20.8)	0.785
Diabetes mellitus	2 (3.6)	3 (12.5)	0.161
Smoking	22 (40.0)	6 (25.0)	0.200
**Clinical symptoms and signs, number (%)**			
Headache	18 (32.7)	8 (33.3)	0.958
Stroke	34 (61.8)	12 (50.0)	0.327
Mood disturbance	15 (27.3)	7 (29.2)	0.863
Cognitive impairment	11 (20.0)	9 (37.5)	0.100
**White matter changes, number (%)**			
Total	53 (96.4)	24 (100.0)	>0.999
Frontal	52 (94.5)	23 (95.8)	>0.999
Parieto-occipital	53 (96.4)	24 (100.0)	>0.999
Anterior temporal	37 (67.3)	6 (25.0)	0.001
External capsule	35 (63.6)	12 (50.0)	0.256
Infratentorial	9 (16.4)	6 (25.0)	0.369
Basal ganglia	37 (67.3)	16 (66.7)	0.958
**Cerebral microbleeds, number (%)**			
Total	22/39 (56.4)	12/16 (75.0)	0.197
Infratentorial	12/39 (30.8)	9/16 (56.3)	0.077
Deep	21/39 (53.8)	11/16 (68.8)	0.309
Lobar	13/39 (33.3)	6/16 (37.5)	0.768

CADASIL, cerebral autosomal dominant arteriopathy with subcortical infarcts and leukoencephalopathy; SD, standard deviation

Table 3 presents the ARWMC scores and MARS. The median ARWMC scores of all patients with CADASIL were high in the frontal and parieto-occipital regions (3 [2–3]), intermediate in the anterior temporal region, external capsule, and basal ganglia (1 [0–2]); and relatively low in the infratentorial region (0 [0–0]). The ordinal logistic regression analysis showed that the cysteine-involving mutation was an independent predictor of the severity of ARWMC scores in the anterior temporal area (proportional odds ratio: 9.7, 95% confidence interval: 2.9–32.6, p < 0.001). Cysteine-involving mutations did not affect the ARWMC scores of the other brain regions.

**Table 3:** ARWMC scores and MARS of patients with CADASIL according to cysteine mutation type.

	Cysteine-involving	Cysteine-sparing	OR (95% CI)	p-value*
**ARWMC scores, median (IQR)**				
Frontal	3 (2–3)	3 (2–3)	1.99 (0.68–5.85)	0.211
Parieto-occipital	3 (2–3)	3 (1–3)	1.88 (0.61–5.85)	0.275
Anterior temporal	1 (0–2)	0 (0–0.75)	9.70 (2.89–32.6)	<0.001
External capsule	1 (0–2)	0.5 (0–2)	1.67 (0.66–4.27)	0.281
Infratentorial	0 (0–0)	0 (0–0.75)	0.54 (0.15–1.91)	0.337
Basal ganglia	1 (0–2)	1 (0–2)	0.92 (0.37–2.30)	0.867
**MARS, median (IQR)**				
Infratentorial	0 (0–1)	1 (0–2.5)	0.54 (0.20–1.41)	0.206
Deep	1 (0–4)	2.5 (0–6)	0.64 (0.24–1.68)	0.361
Lobar	0 (0–2)	0 (0–4.75)	0.77 (0.28–2.14)	0.622

* Adjusted for covariates including age, hypertension, diabetes mellitus, and smoking.

ARWMC, age-related white matter change; CADASIL, cerebral autosomal dominant arteriopathy with subcortical infarcts and leukoencephalopathy; IQR, interquartile range; MARS, microbleed anatomical rating scale; OR, odds ratio.

**Fig 1:**
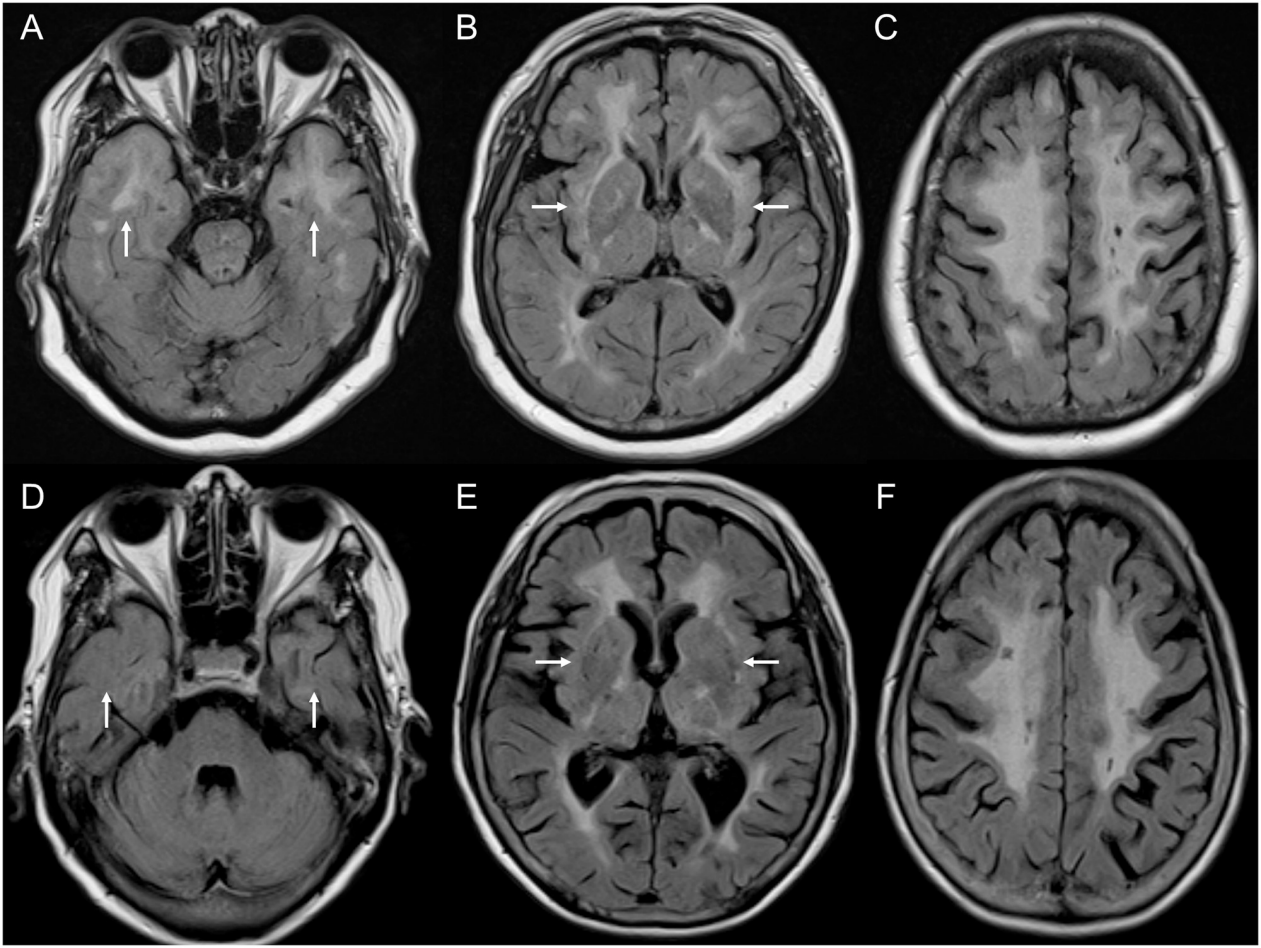
Representative brain MRIs. MRIs of patients with CADASIL and cysteine-involving (C174R, 66/F, A-C) or cysteine-sparing (R75P, 67/F, D-F) NOTCH3 mutations are shown. Axial fluid attenuation inversion recovery (FLAIR) images showing similar diffuse frontal, parietal subcortical white matter changes (C, F), and external capsule involvements (B, E). However, anterior temporal lobe involvement is seen only in the patient with a cysteine-involving NOTCH3 mutation (A) and is absent in the patient with a cysteine-sparing NOTCH3 mutation (D). CADASIL, cerebral autosomal dominant arteriopathy with subcortical infarcts and leukoencephalopathy; MRI, magnetic resonance imaging.

MARS analysis showed that cysteine-involving mutations did not affect the numbers of microbleeds in each brain region between the groups. Fig 1 shows representative brain MRI findings from patients with CADASIL and cysteine-involving or cysteine-sparing mutations.

## Discussion

This study analyzed and compared clinical and imaging characteristics in patients with CADASIL between those with cysteine-sparing mutations and those with cysteine-involving mutations. Our results revealed that patients with CADASIL and cysteine-sparing NOTCH3 mutations had less involvement of the anterior temporal lobes in brain MRIs, although both groups showed similar clinical characteristics. Whether cysteine-sparing NOTCH3 mutations cause CADASIL remains controversial [15]. However, we showed that cysteine-sparing NOTCH3 mutations manifested similar demographics, clinical symptoms, and signs in patients, which was comparable to the cysteine-involving mutations. We also showed that except for a tendency to have less involvement of anterior temporal lobes, patients with CADASIL and cysteine-sparing mutations had brain MRI features similar to those patients with cysteine-involving mutations. These findings are consistent with those of a previous systematic review of cysteine-sparing NOTCH3 mutations in patients with CADASIL [20]. In addition, after adjusting for age, hypertension, diabetes mellitus, and smoking, patients with CADASIL and cysteine-involving mutations had a 9.7-fold higher odds of having a more severe score in the anterior temporal lobe (i.e. ARWMC score 1–3 versus ARWMC score 0) than that of patients with CADASIL and cysteine-sparing mutations.

A previous brain MRI study reported the spatial distribution of white matter changes in patients with CADASIL, grading the severity as high in the frontal, parietal, anterior temporal cortex, and external capsule [21]. These findings are concordant with our results indicating high ARWMC scores in the frontal and parieto-occipital regions; intermediate in the anterior temporal cortex, external capsule, and basal ganglia; and relatively low in the infratentorial region. In our study, the frequency of temporal lobe hyperintensity of all patients with CADASIL was 54%, lower than that of 89–95% in reports of Caucasian patients [22, 23]. However, the frequency of temporal lobe involvement in brain MRI was reportedly relatively low in East Asian countries, in which 71% of Japanese patients with CADASIL had anterior temporal lobe involvement [12] and 46% of Chinese patients had temporal pole involvement [13], compared to only 43% of Taiwanese patients [9]. Among Korean patients, studies have reported 54% and 20% of patients with CADASIL with temporal pole T2 hyperintensities in brain MRI [7, 8]. Our review of the literature and our findings showed that patients with CADASIL and cysteine-involving R544C or cysteine-sparing R75P mutations tended to present less temporal pole involvement in brain MRI compared to other patients [7–9, 12]. The frequencies of R544C and R75P mutations are relatively high in East Asian countries, but, to our knowledge, these mutations have not been reported in Caucasian countries, which might explain the low frequency of anterior temporal lobe involvement in Asian patients with CADASIL (Table 4).

**Table 4:** Frequency of anterior temporal lobe T2 hyperintensities in brain MRI and R544C and R75P NOTCH3 missense mutations in the present study and other studies.

CADASIL series, number (%)	Anterior temporal lobe involvement	R544C	R75P	Country
**Markus et al, 2002**	41/46 (89)	0/48 (0)	0/48 (0)	UK
**Kim et al, 2006**	14/26 (54)	5/27 (19)	16/27 (59)	Korea
**Choi et al, 2006**	4/20 (20)	15/20 (75)	2/20 (10)	Korea
**Lee et al, 2009**	9/21 (43)	10/21 (48)	0/21 (0)	Taiwan
**Wang et al, 2010**	22/48 (46)	0/57 (0)	1/57 (2)	China
**Ueda et al, 2015**	36/51 (71)	0/70 (0)	8/70 (11)	Japan
**Present study**	43/79 (54)	12/79 (15)	18/79 (23)	Korea

CADASIL, cerebral autosomal dominant arteriopathy with subcortical infarcts and leukoencephalopathy; MRI, magnetic resonance imaging.

The pathology of patients with CADASIL shows an accumulation of NOTCH3 protein in the vessel walls [24]. Thus, the propensity to form aggregates seems to be relevant to the pathogenesis of NOTCH3 mutations [5]. Wollenweber and Hanecker et al. showed that not only cysteine-involving mutations but also cysteine-sparing mutations (including R75P and D80G) have pro-aggregatory properties with *in vitro* multimerization [25]. They also showed that the other cysteine-sparing NOTCH3 mutations, R61W and R213K, had no multimerization tendency, indicating a polymorphism rather than disease-causing mutation. Therefore, they suggested that cysteine-sparing mutations causing NOTCH3 receptor aggregation lead to a CADASIL phenotype similar to that caused by cysteine-involving NOTCH3 mutations [25]. The different tendencies of temporal pole involvement between CADASIL with cysteine-sparing mutations and that with cysteine-involving mutations might be explained by the temporal pole’s unique convolutional structure and vascularization [26]. If we assume that the temporal pole is less vulnerable to leukoaraiosis than the other brain regions, this might be explained by the different tendencies of NOTCH3 protein accumulation around vascular smooth muscle cells between cysteine-involving and cysteine-sparing mutations. This speculation requires verification in future studies.

The limitations of our study include its retrospective design and the limited analysis of the NOTCH3 gene sequences. We analyzed only the hotspots of NOTCH3 (exons 2–11 and 18) and may have missed pathogenic mutations in non-analyzed exons. In terms of the pathogenicity of novel cysteine-sparing variants, we relied on bioinformatics predictions only and not skin biopsy pathology results. A prospective, large-scale study that can identify pathological findings through skin biopsy would overcome these limitations.

In conclusion, patients with CADASIL and cysteine-sparing NOTCH3 mutations showed less involvement of the anterior temporal lobes in brain MRI compared to those with CADASIL and cysteine-involving NOTCH3 mutations, although both groups showed similar clinical characteristics.

## Supporting information

S1 Table Comparisons of scores between left and right hemispheres. (DOCX)

S1 File (XLSX)
