# Supplementary material for: Clinical and imaging features of patients with cerebral autosomal dominant arteriopathy with subcortical infarcts and leukoencephalopathy and cysteine-sparing NOTCH3 mutations
Source: PLoS One. 2020 Jun 18;15(6):e0234797. doi: 10.1371/journal.pone.0234797 (PMC7302479; doi:10.1371/journal.pone.0234797)
Supplement: S1 Table — (DOCX) [file pone.0234797.s001.docx]

**S1 Table.** **Comparisons of scores between left and right hemispheres**

|  | Left | Right | *p* value |
| --- | --- | --- | --- |
| ARWMC scores, mean ± SD | | | |
| Frontal | 2.41 ± 0.86 | 2.41 ± 0.87 | >0.999 |
| Parieto-occipital | 2.39 ± 0.85 | 2.47 ± 0.73 | 0.549 |
| Anterior temporal | 1.09 ± 1.16 | 1.06 ± 1.15 | 0.890 |
| External capsule | 0.84 ± 1.01 | 0.99 ± 0.98 | 0.338 |
| Infratentorial | 0.27 ± 0.65 | 0.30 ± 0.69 | 0.722 |
| Basal ganglia | 1.03 ± 0.97 | 1.11 ± 0.99 | 0.571 |
| MARS, mean ± SD | | | |
| Infratentorial | 1.18 ± 1.99 | 1.02 ± 1.95 | 0.664 |
| Deep | 2.96 ± 4.00 | 3.13 ± 4.08 | 0.832 |
| Lobar | 2.60 ± 6.84 | 2.67 ± 6.35 | 0.954 |

ARWMC, age-related white matter change; MARS, microbleed anatomical rating scale; SD, standard deviation.
